# Transcriptome and Metabolome Comparison of Smooth and Rough *Citrus limon* L. Peels Grown on Same Trees and Harvested in Different Seasons

**DOI:** 10.3389/fpls.2021.749803

**Published:** 2021-10-08

**Authors:** Hong-ming Liu, Chun-rui Long, Shao-hua Wang, Xiao-meng Fu, Xian-yan Zhou, Jia-mei Mao, Hong-xia Yang, Yu-xia Du, Jin-xue Li, Jian-qiang Yue, Fa-guang Hu

**Affiliations:** Institute of Tropical and Subtropical Cash Crops, Yunnan Academy of Agricultural Sciences, Baoshan, China

**Keywords:** autumn flowering fruit, transcriptome, metabolome, peel roughening, phytohormone signaling, cell division

## Abstract

**Background:** Farmers harvest two batches fruits of Lemons (*Citrus limon* L. Burm. f.) i.e., spring flowering fruit and autumn flowering fruit in dry-hot valley in Yunnan, China. Regular lemons harvested in autumn have smooth skin. However, lemons harvested in spring have rough skin, which makes them less attractive to customers. Furthermore, the rough skin causes a reduction in commodity value and economical losses to farmers. This is a preliminary study that investigates the key transcriptomic and metabolomic differences in peels of lemon fruits (variety Yuning no. 1) harvested 30, 60, 90, 120, and 150 days after flowering from the same trees in different seasons.

**Results:** We identified 5,792, 4,001, 3,148, and 5,287 differentially expressed genes (DEGs) between smooth peel (C) and rough peel (D) 60, 90, 120, and 150 days after flowering, respectively. A total of 1,193 metabolites differentially accumulated (DAM) between D and C. The DEGs and DAMs were enriched in the mitogen-activated protein kinase (MAPK) and plant hormone signaling, terpenoid biosynthesis, flavonoid, and phenylalanine biosynthesis, and ribosome pathways. Predominantly, in the early stages, phytohormonal regulation and signaling were the main driving force for changes in peel surface. Changes in the expression of genes associated with asymmetric cell division were also an important observation. The biosynthesis of terpenoids was possibly reduced in rough peels, while the exclusive expression of cell wall synthesis-related genes could be a possible reason for the thick peel of the rough-skinned lemons. Additionally, cell division, cell number, hypocotyl growth, accumulation of fatty acids, lignans and coumarins- related gene expression, and metabolite accumulation changes were major observations.

**Conclusion:** The rough peels fruit (autumn flowering fruit) and smooth peels fruit (spring flowering fruit) matured on the same trees are possibly due to the differential regulation of asymmetric cell division, cell number regulation, and randomization of hypocotyl growth related genes and the accumulation of terpenoids, flavonoids, fatty acids, lignans, and coumarins. The preliminary results of this study are important for increasing the understanding of peel roughness in lemon and other citrus species.

## Introduction

Lemon (*Citrus limon* L. Burm. f.) is an important commercial and nutritious fruit that is a member of the Rutaceae family. It originated in Asia (likely in the Punjab region of Pakistan and India, China, or Myanmar); however, it is now cultivated throughout the world in tropical and subtropical regions and consumed globally (Gulsen and Roose, 2001). China is the top producer of lemons, with 2,405.9 thousand tonnes produced only in 2016 (Food and Agriculture Organization, 2017). Being a dominant agricultural product of mountainous regions of southwest China, it has become a major industry. Among the southwestern Chinese areas, Yunnan is the major lemon producer. The unique geographical and climatic conditions of the dry-hot valley region of the Nujiang River on the southwest border of Yunnan support the annual flowering and fruiting of lemon trees. Particularly, the farmers in this region harvest two batches fruits of lemons i.e., spring flowering fruit and autumn flowering fruit similar to its cultivation in Punjab, India (Kaur et al., 2019). These features enable the farmers in Yunnan to have relatively higher profitability as compared with farmers in other regions of China and neighboring countries.

In a regular season, the trees flower in spring, and the lemons are harvested in autumn. These fruits have smooth skin and a high commercial value. However, the fruits that are harvested in spring have different characteristics, such as being heavier (158.9 g as compared with 128.75 g) and having a thicker peel (0.89 cm as compared with.55 cm) and rough surface/skin. According to our pilot studies, the yield of the smooth peel fruits is higher (38.2 kg/plant) than that of the roughly peeled fruits with a rough peel (27.55 kg/plant). Furthermore, the edible rate of the smooth-peeled lemon fruits is greater (0.66) than that of the rough-peeled lemon fruits (0.44) (un published data). Such characteristics make the lemon fruits harvested in spring less attractive to customers (Sinanta, 2013; Polat, 2018; Klimek-Szczykutowicz et al., 2020). Particularly, the rough skin reduces its commodity value and, ultimately, causes losses to the farmers (Kaur et al., 2019). Understanding the molecular mechanisms of smooth and rough skin formation can ensure the farmers with a higher economic output.

The rough skin in citrus, e.g., Satsuma Mandarin (*Citrus unshiu* Marc.), is known as peel roughening disorder (RD), rough peel disorder, peel roughness, or rind roughness (Food and Agriculture Organization, 2017). Very limited information is available on possible causes and molecular mechanisms governing RD. In other citrus fruits, limited literature explains that some of the reasons for RD include changes in endogenous hormones (cytokinin-and gibberellin), soil, air humidity, and rootstock (Erner et al., 1975, 1976). Recent studies explored the metabolome profile of Sasuma Mandrin peels with RD. The authors reported that sugars, organic acids, and amino acids and derivatives were the main metabolites that were differentially accumulated. Particularly, the amino acids and derivatives accumulated in higher quantities in the RD peels (Food and Agriculture Organization, 2017; Lu et al., 2017). The authors of a latest study only considered the peels of fruits at 170 days after full bloom, thus, leaving a knowledge gap between the early developmental stages of the citrus peels (Lu et al., 2017). Another study on the development of citrus peels has reported the differential accumulation of organic acids and derivatives, polyphenols, amino acids and derivatives, flavones, and nucleotides and derivatives (Wang et al., 2020). In recent years, the development in sequencing and metabolite profiling has enabled the authors to explore large-scale transcriptomic and metabolomic signatures of differential phenotypes (Kell and Oliver, 2016). In recent studies, these techniques have greatly supplemented the understanding of the response of *C. limon* and other citrus species against biotic and abiotic stresses (Ramsey et al., 2020; Chin et al., 2021; Zhou et al., 2021).

This study is a first attempt to explore the key transcriptomic and metabolomic differences in both smooth and rough peel types in *C. limon* fruits grown on the same trees and harvested in different seasons, i.e., spring and autumn. We performed Illumina high-throughput transcriptome sequencing and employed widely targeted based metabolome profiling techniques. This study will yield a preliminary understanding of changes in the expression of genes in multiple pathways in the two types of peel of lemons that matured in different seasons.

## Results

### Transcriptomic Response *Citrus limon* Fruits With C and D

**FIGURE 1:**
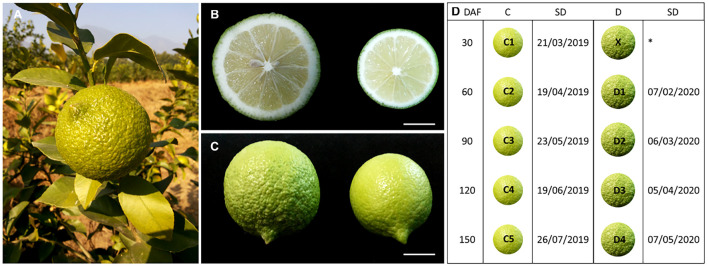
**(A)** Large fruit with thick skin, **(B)** Comparison of (left) rough- and (right) smooth- skinned lemon fruits (cut), **(C)** whole fruit, and **(D)** information on the sampling dates for C; control and D) representation of the experimental fruit groups. The circular images are representations of the fruit peels and do not reflect the size based on different DAF. DAF, days after flowering; SD, sampling date. ^∗^ indicates that fruit samples were not collected because of the seasonal effect. Scale bar = 1 cm.

**FIGURE 2:**
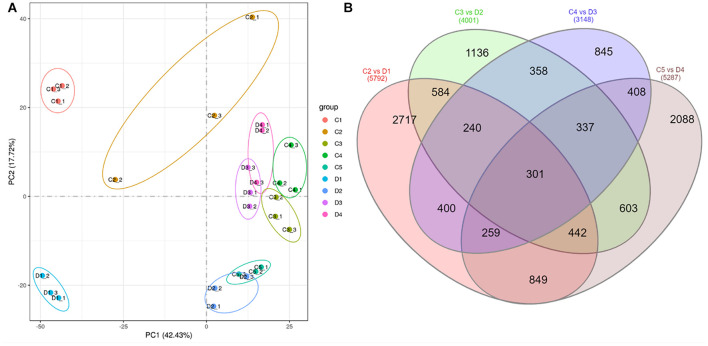
**(A)** Principal component analysis of treatments of C (control group) and D (experiment group) of *Citrus limon* fruit peels, and **(B)** Venn diagram of differentially regulated genes by different treatment comparisons. C1, C2, C3, C4, and C5 represent the samples collected 30, 60, 90, 120, and 150 days after flowering. D1, D2, D3, and D4 represent the samples collected 60, 90, 120, and 150 days after flowering. The numbers 1, 2, and 3 with each treatment represent the three replicates.

The transcriptome of the two types of peel of *C. limon* [smooth (C) and rough (D)] harvested 30, 60, 90, 120, and 150 days after bloom (Figure 1) was sequenced using a Novaseq (Illumina, San Diego, CA, United States) platform. The sequencing of 27 libraries resulted in 40,211,564 to 47,275,744 and 39,568,768 to 46,284,626 raw and clean reads, respectively. The Q20, Q30, and GC% was >98, >94, and >44%, respectively (Supplementary Table 1). The gene expression level (FPKM) of fruit peels harvested after 30 days was high both for C and D (Supplementary Figure 1A). The Pearson’s correlation coefficient between the samples ranged from.632 to.98 (Supplementary Figure 1B). Principal component analysis (PCA) showed that the treatment replicates grouped together, indicating the reliability of sampling (Figure 2A).

### Differential Gene Expression in *Citrus limon* Fruit Peels

#### Common Differentially Expressed Genes in All Time Points

The differential comparison between C and D harvested at different times resulted in the identification of 5,792, 4,001, 3,148, and 5,287 differentially expressed genes (DEGs) in C2 vs. D1, C3 vs. D2, C4 vs. D3, and C4 vs. D5, respectively (Figure 2B). Of these, 301 genes were differentially expressed in all the comparisons of peels harvested after 60, 90, 120, and 150 days (Figure 2B and Supplementary Table 2). By looking at the log 2 fold change values of these DEGs, we found that 27 genes were downregulated in D at all the time points. These DEGs included protein NRT1/PTR FAMILY 6.1, MDIS1-interacting receptor-like kinase 2, phosphoenolpyruvate carboxykinase (ATP), CYP82D47, CYP89A2, aquaporin NIP1-2, aquaporin AQPAn.G, shikimate O-hydroxycinnamoyltransferase-like, and coumarin 8-geranyltransferase 1 2C chloroplastic-like. On the contrary, only three genes were upregulated in D as compared with C in all the time points; a xyloglucan galactosyltransferase XLT2 (*LOC18033075*), a putative glycosyltransferase 7 (*LOC18037353*), and a L-type lectin-domain containing receptor kinase S4 (*LOC18045464*).

Interestingly, 124 genes were upregulated in D as compared with C after 60, 90, and 120 days but downregulated after 150 days. Prominently, these included cell wall-related genes, calcium signaling-related genes, and a large number of uncharacterized genes. In these 124 genes, we also found ethylene-responsive transcription factor (TF) (ERF003, *LOC18053793*), GPI-anchored protein LLG1 (*LOC18055492*), nitrate regulatory gene 2 (*LOC18042616*), protein GRAVITROPIC IN THE LIGHT 1 (GIL1, *LOC18046532*), syntaxin-related protein KNOLLE (*LOC18049273*), TF bHLH62 (*LOC18033163*), and zinc transporter 1 (*LOC18032278*) (Supplementary Table 2). Ninety-eight genes were downregulated in D as compared with C after 60, 90, and 120 days, and upregulated in D after 150 days, suggesting an important role in peel phenotype development. These DEGs included probable glutathione-S-transferases, WRKY4, WRKY28, potassium channel AKT1, hsp70, oxygen-dependent coproporphyrinogen-III oxidase 2C chloroplastic, PAP-specific phosphatase HAL2-like, pyruvate decarboxylases, and alcohol dehydrogenase 1s (Supplementary Table 2).

#### Differentially Expressed Genes Expressed Exclusively in Rough-Peeled *Citrus limon*

A total of 34, 26, 12, and 15 genes were exclusively expressed in D peels harvested after 60, 90, 120, and 150 days (Supplementary Table 3). Interestingly, these DEGs were also specific to the harvesting time. The functional exploration of these genes suggested their roles in multiple pathways. For example, genes that were expressed in the D peels harvested after 60 days included ethylene responsive AIL1TF, auxin-induced protein 15 A, probable indole-3-pyruvate monooxygenase YUCCA4 (Cheng et al., 2006), cytokinin riboside 5’-monophosphate phosphoribohydrolase LOG1 (Kuroha et al., 2009), and F-box protein SNE. The other genes solely expressed in the D peels harvested after 60 days were related to the thalianol pathway (BAHD acyltransferase, *LOC18052907*), fatty acid elongation (elongation of fatty acids protein 3-like and A-like (Quist et al., 2009), *LOC18040630* and *LOC112098405*), carotenoid cleavage (probable carotenoid cleavage dioxygenase 4, *LOC18038224*), sugar partitioning (TF CSA, *LOC18045199*), and tyrosine decarboxylation (tyrosine decarboxylase1, *LOC18037575* and *LOC18037081* and tyrosine decarboxylase 4, *LOC18037218*) (Supplementary Table 3).

Genes that were expressed in the D peels harvested after 90 days were related to ethylene signaling, cell wall biogenesis, lignin degradation and detoxification of lignin-derived products, polyamine back conversion, demethylesterification of pectin, and light control of development (probably due to the expression of protein FAR1-RELATED SEQUENCE 5-like in D). We also observed the exclusive expression of protein SHORT-ROOT (SHR, *LOC18048780*) in the D peels harvested after 90 days. While, in the case of D peels harvested after 120 days had an exclusive expression of two cationic amino acid transporters (LOC18049397 and LOC18050423), ERF13 (LOC18047945), methanol O-anthraniloyltransferase (*LOC18050768*), and protein ubiquitination-related gene (putative RING-H2 finger protein ATL21A, *LOC18048562*). These expression changes suggest that at this stage, the rough peels of D have increased amino acid transport, increased biosynthesis of methyl anthranilate (Wang and Luca, 2005), and ethylene related activities. The D peels harvested after 150 days had an exclusive expression of ERF096, ERF13, anthocyanidin 3-O-glucosyltransferase 2, cinnamoyl-CoA reductase 2, gibberellin 2-beta-dioxygenase 1, LOB domain-containing protein 12 (LBD12), lysine histidine transporter-like 8, metalloendoproteinase 1-MMP, probable S-adenosylmethionine-dependent methyltransferase, MYB78, and UDP-glycosyltransferase 74E2. From these exclusive expressions, it can be concluded that ethylene signaling and biosynthesis, regulation of gibberellins, changes in anthocyanin, carotenoid biosynthesis, and amino acid transport are playing some roles in the observed phenotype (Supplementary Table 3).

### Differentially Expressed Genes Expressed Exclusively in Smooth-Peeled *Citrus limon*

A total of 62, 16, 17, and 19 genes were exclusively expressed in C peels harvested after 60, 90, 120, and 150 days, respectively. The genes that were expressed in C2 were related to flavonoid biosynthesis pathway, flowering-related pathway, carbohydrate metabolism, fruit ripening (CYP71A1), calcium homeostasis, and heat stress. The genes expressed in C3 were related to reactive oxygen species production, auxin signaling, cell number regulation, ethylene signaling, amino acid biosynthesis, and stomata differentiation.

Similar to C2 and C3, we noticed the expression of ethylene related genes (e.g., 1-aminocyclopropane-1-carboxylate oxidase homolog 1), flavonoid biosynthesis pathway, carotenoid biosynthesis (geranylgeranyl pyrophosphate synthase 7 chloroplastic-like, higher GGP biosynthesis means higher carotenoid biosynthesis), cuticle development (probable lysophospholipase BODYGUARD 3), fruit ripening (protein PELPK1, (Rashid, 2014)), and organ abscission [TF RAX3 (Pei et al., 2016)] in C4. The exclusively expressed genes in C5 as compared with D4 were mostly related to cell-wall, flavonoid biosynthesis, stomata closure related genes, and anthocyanin biosynthesis related genes and TFs, e.g., myb-related protein 308 (Supplementary Table 3).

### Enrichment of Differentially Expressed Genes in Kyoto Encyclopedia of Genes and Genomes Pathways

The DEGs in C2 vs. D1 were significantly enriched in three pathways i.e., MAPK signaling pathway-plant, DNA replication, and glyoxylate and dicarboxylate metabolism. In addition to MAPK signaling pathway-plant, the DEGs in C3 vs D2 were enriched in amino sugar and nucleotide sugar metabolism, pentose and glucuronate interconversion, phenylpropanoid biosynthesis, and ubiquinone and other terpenoid-quinone biosynthesis pathways. With the increase in the number of days after flowering, i.e., 120 days after flowering (DAF), the DEGs (in C3 vs. D4) were enriched in the terpenoid backbone biosynthesis, phenylpropanoid biosynthesis, amino sugar and nucleotide sugar metabolism, flavonoid biosynthesis, stilbenoid, diarylheptanoid and gingerol biosynthesis, and phenylalanine, tyrosine, and tryptophan biosynthesis pathways. The DEGs in C4 vs. D5 were significantly enriched in the ribosome pathway (Supplementary Figure 2). The enrichment results suggest the changes of hormonal and developmental signaling, sugar and amino acid contents, and pigment and scent compounds during lemon peel development at 60 and 90 days. In the latter stages of lemon peel development, the biosynthesis of phenylpropanoids, terpenoids, phenylalanine, tyrosine and tryptophan, and amino sugars was differentially regulated. Thus, below, we present pathway-specific transcriptomic changes in C and D to explore more about key changes in both peel types.

#### Regulation of Mitogen-Activated Protein Kinase and Phytohormone Signaling

**FIGURE 3:**
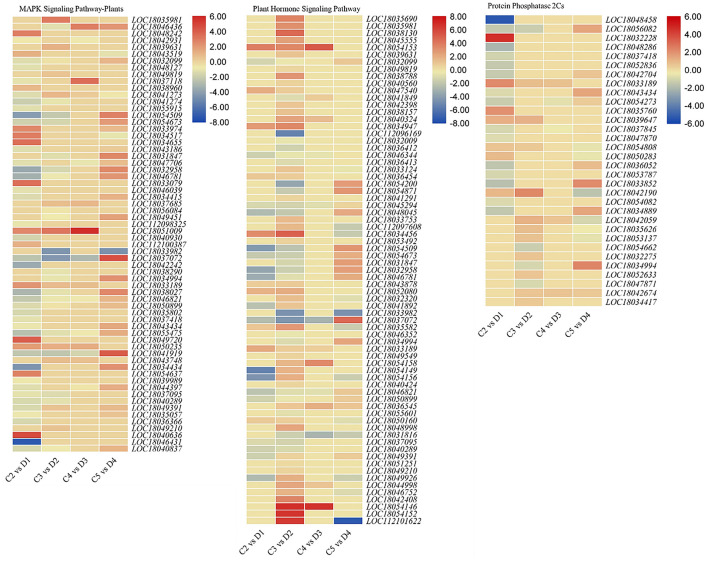
Heatmaps of log2 FC of differentially expressed genes (DEGs) enriched in the MAPK and plant hormone signaling pathways in treatment comparisons between C and D of *C. limon.* C2, C3, C4, and C5 represent samples collected 60, 90, 120, and 150 days after flowering. D1, D2, D3, and D4 represent samples collected 60, 90, 120, and 150 days after flowering. The numbers 1, 2, and 3 with each treatment represent the three replicates. Gene IDs are consistent with the gene descriptions given in Supplementary Table 4.

Sixty-four and 71 DEGs were differentially regulated between both peel types in the MAPK and plant hormone signaling pathways, respectively (Figure 3 and Supplementary Table 4). We observed that abscisic acid (ABA) receptor PYLs were differentially regulated between the C and D peels. PYL4s (*LOC18039631* and *LOC18043519*) and PYL9 (*LOC18049819*) were upregulated, while PYL8s (*LOC18032099* and *LOC18048127*) were downregulated in D as compared with C. We also observed the decreased expression of PP2C genes in D1 and D2 (Figure 3), and the downregulation of ethylene related genes in D (60 and 90 DAF) as compared with C, i.e., EIN1s (EIN3-binding F-box protein 1), ethylene insensitive 3-like 1 (EIN3) and EIN4, ethylene response sensor 1, and ethylene-responsive TF 1Bs (Supplementary Table 4). This suggests increased ethylene insensitivity in D1 and D2 as compared with C2 and C3, respectively. MAPKK2, MAPKK9, PRP1s, CML29, CML45, respiratory burst oxidase homolog protein B (RBOHB), RBOHD, serine/threonine-protein kinases (CTR1, SAPK2, SAPK3, SRK2E, and SRK2I), WRKY22, and WRKY24 were downregulated in D as compared with C, while other genes, i.e., endochitinases, MAPKKK18, and nucleoside diphosphate kinase 1 were upregulated in D as compared with C (Figure 3 and Supplementary Table 4).

The DEGs annotated in auxin signaling and transport were differentially expressed in the 60- and 90-DAF-harvested fruits with C and D. Among the auxin-responsive proteins, AUX22, AUX28, IAA1, IAA29, SAUR32, and SAUR36 were downregulated, while IAA27, SAUR50, and SUAR72-like were upregulated in D as compared with C. Similar to the MAPK signaling pathway, the ethylene related genes were downregulated in D as compared with C. A single gibberellin signaling-related gene (DELLA protein GAIP-B) was upregulated in D2 as compared with C3. Genes related to brassinosteroid (and resultingly), e.g., protein BRASSINAZOLE-RESISTANT 1, XTH22, and XTH24, were upregulated in D as compared with C, suggesting a possible role in thicker peels (Figure 3 and Supplementary Table 4).

#### Regulation of Other Important Pathways

Eighteen and 22 DEGs were enriched in ubiquinone and other terpenoid-quinone biosynthesis and terpenoid biosynthesis pathways, respectively. Interestingly, most of the DEGs in ubiquinone and other terpenoid-quinone biosynthesis pathway were downregulated in D as compared with C in almost all the treatment comparisons; prominently 4-coumarate–CoA ligase-like genes and coumarin 8-geranyltransferase 1 (chloroplastic-like). These expression changes suggest reduced terpenoid biosynthesis in D as compared with C. This is consistent with the downregulation of all the genes (except 3-hydroxy-3-methylglutaryl-coenzyme A reductase 1) in D (harvested 120 DAF) as compared with C (Supplementary Table 4). The enrichment of phenylpropanoid biosynthesis pathway showed reduced expression genes related to anthocyanidin and 4-coumarate in D. Among other genes, shikimate O-hydroxycinnamoyltransferases, caffeic acid 3-O-methyltransferases, caffeoyl-CoA O-methyltransferase, and cytochrome p450s were largely downregulated in D as compared with C. A large number of peroxidases (17) showed a variable expression between both peel types. The expression trend of genes enriched in phenylalanine, tyrosine, and tryptophan biosynthesis, and flavonoid biosynthesis was quite similar to that of phenylpropanoid pathway-related genes (Supplementary Table 4). The ribosome pathway-related DEGs were only expressed in the C5 vs. D4 treatment comparison and showed log2 fold changes values < 1 (with few exceptions), indicating an increased transcriptional regulation in D4 as compared to C5 (Martinez-Seidel et al., 2020).

### Quantitative Real-Time PCR Analysis

**FIGURE 4:**
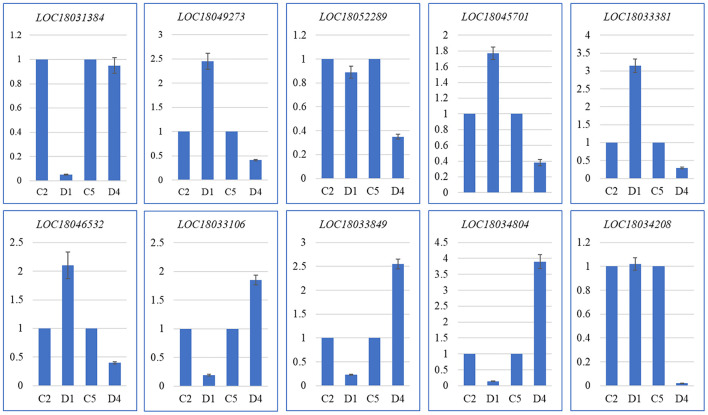
Quantitative real-time (qRT)-PCR analysis of selected DEGs in C and D of *C. limon.* D1 and C2, and D4 and C5 represent the samples collected 60 and 150 days after flowering, respectively. The *Y*-axis represents relative gene expression (mean of three replicates) and the *X*-axis represents *C. limon* peels harvested at different days after flowering. The error bars represent SD.

The quantitative expression of 10 *C. limon* genes in D1 and C2, and D4 and C5 was confirmed by quantitative real-time (qRT)-PCR analysis. The expression profiles were similar to those observed in the RNA-sequencing (Figure 4 and Supplementary Tables 2–4). Thus, these expression profiles confirm that the transcriptome sequencing results are valid and reliable.

### Metabolomic Response of *Citrus limon* Fruit Peels

**FIGURE 5:**
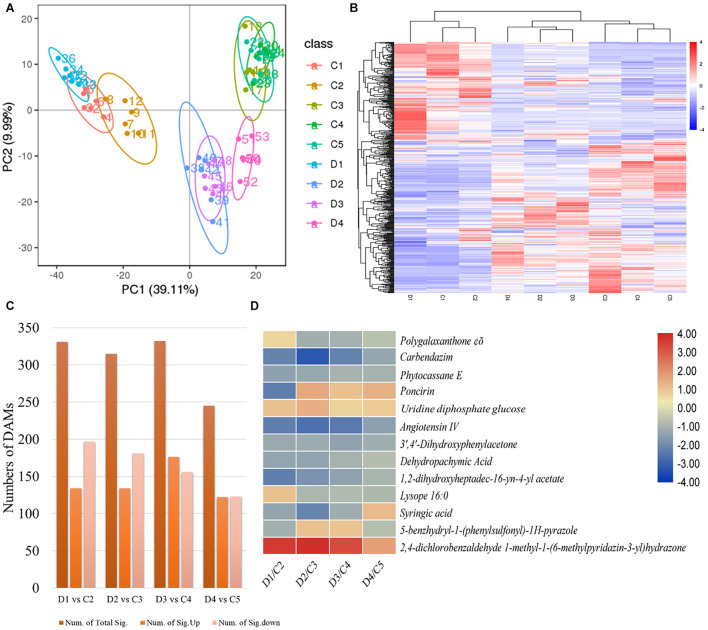
**(A)** Principal component analyses, **(B)** heatmap of the differentially accumulated metabolites (DAMs), **(C)** number of DAMs in each treatment comparison, and **(D)** common accumulated metabolites in C (control group) and D (experiment group) of *Citrus limon* fruit peels. C1, C2, C3, C4, and C5 represent the samples collected 30, 60, 90, 120, and 150 days after flowering. D1, D2, D3, and D4 represent the samples collected 60, 90, 120, and 150 days after flowering. The numbers 1, 2, and 3 with each treatment represent the three replicates. The bar on the side shows the scale of metabolite intensities from lowest (blue) to highest (red). **(C)** Summary of metabolites detected in each treatment comparison between **(C,D)**. **(D)** Heatmap of the log2 fold change values of the commonly detected DAMs in both types of peels in all days after flowering. The scale bar on the side represents the lowest (blue) to highest (red) log2 fold change values of the DAMs.

The PCA based on metabolites showed grouping of treatments of the same experimental group, indicating that the results are reliable (Figure 5A). We identified a total of 1,083 metabolites in the *C. limon* fruit peels (Figure 5B and Supplementary Table 5). For treatment comparisons D1 vs. C2, D2 vs. C3, D3 vs. C4, and D4 vs. C5, there were 331, 314, 332, and 245 metabolites that were significantly differentially accumulated (DAMs), respectively (Figure 5C). Thirteen common metabolites were accumulated in both peels collected after all the sampling dates (Figure 5D).

The DAMs in the treatment comparisons were enriched in multiple pathways (Supplementary Figure 3). Particularly, we observed that the DAMs in D2 vs. C1 were enriched in 36 pathways such as those observed in transcriptome-based KEGG pathway enrichment. Interestingly, we observed the enrichment of DAMs in the flavonoid biosynthesis, plant hormone signal transduction, anthocyanin biosynthesis, and amino acid related pathways. The DAMs in D2 vs. C3, D3 vs. C4, and D4 vs. C5 were enriched in 33, 20, and 25 pathways, respectively. The results of the transcriptome-based KEGG pathway enrichment analysis showed that the DAMs were enriched in some of the pathways (Supplementary Figures 2, 3). These observations confirm the transcriptome-based results that the *C. limon* peels harvested after 60 and 90 days in spring and autumn have a differential regulation of hormone signaling, amino sugar and amino acid-related pathways, and pigment related pathways. While in the latter DAF, the *C. limon* peels have a differential regulation of the phenylpropanoid and terpenoid, and tyrosine and tryptophan biosynthesis pathways.

### Differential Metabolite Accumulation in C and D

A total of 331 metabolites were differentially accumulated in D1 vs C2; 197 were downregulated and 134 were upregulated in D1 as compared with C2. The downregulated metabolites belong to fatty acids and conjugates, flavones and flavonols, flavanons, amino acids, peptides and analogs, benzoic acid and derivatives, carbohydrates, and terpenoids (Supplementary Table 5). The metabolites that were increasingly accumulated in D1 as compared with C2 mostly belonged to flavonoids and flavonols, and amino acids, peptides, and analogs. In the case of the treatment comparison D2 vs. C3, 314 metabolites accumulated differentially; the content of 181 and 133 metabolites was decreased and increased, respectively, in D2 as compared with C3. The metabolites with decreased content in D2 were classified as fatty acids and conjugates, fatty alcohols, fatty amides, fatty esters, glycerophosphocholines, flavonoids, isoprenoids, and steroids. Additionally, we found lignans, terpene lactones, amino acids, peptides and analogs, sesquiterpenoids, and monoterpenoids in reduced quantities in D2 as compared with C3. On the contrary, flavonoids, sterols, isoprenoids, and several fatty acids and conjugates were increasingly accumulated in D2 as compared with C3. The differentially accumulated metabolites in D3 vs. C4 were classified as alcohols and polyols, benzene and substitutive derivatives, carboxylic acids and derivatives, coumarins, fatty acyls, flavonoids, carbohydrates, and terpenoids and derivatives. A similar class of compounds was differentially accumulated in the treatment comparison of the *C. limon* peels collected 150 DAF i.e., D4 vs. C5 (Supplementary Table 5).

## Discussion

Improving aesthetics for preference by consumers is one of the major tasks of fruit breeders across the world. Fruit aesthetic, along with freshness and flavor, is the major driver of consumer preference in citrus species (Gao et al., 2011). *C. limon* fruits are harvested twice a year i.e., spring and autumn (Kaur et al., 2019). The fruits that are harvested in spring have a rough peel surface, which leads toward reduced aesthetic value, and consumers do not prefer it over those with smooth skin. This, in turn, results in reduced income. This is a preliminary study to explore the transcriptomic and metabolomic differences that exist between both peel types grown on the same trees but in different seasons. The differential development of D and C fruits probably causes the early changes in their respective peels. Predominantly, in the early stages (60 and 90 days in this study), phytohormonal regulation and signaling were the main driving force for the changes in peel surface. We say this because the exclusive genes expressed in D were mainly related to hormone signaling (Supplementary Table 3). Particularly, the observation that genes involved in ethylene signaling could be the leading cause of color differences in peels; the increased expression of ETR2 and decreased expression of EIN1s, EIN3, EIN4, ethylene response sensor 1, and ethylene-responsive TF 1Bs. It is known that ethylene and ABA balance causes abscission in citrus fruits, while ethylene alone causes peel degreening (Goldschmidt et al., 1993). This is more relevant to the color of the peel rather than rough development. Also, ethylene has long been used externally for citrus fruits to promote coloring (Carmona et al., 2014). If it had any roughening effects on the skin, its use might have been abandoned. Thus, it could not be directly associated with peel roughness in D. Specific characterization studies targeting ethylene, lemon peel color, and the effect of carotenoid should elaborate more on color differences between both peel types. The MAPK and plant hormone signaling pathways play essential roles in plant development (Adams-Phillips et al., 2004; Jagodzik et al., 2018). Particularly, the regulation of both signaling pathways in the C and D collected after 60 and 90 days indicates their prominent role in peel roughening or smoothening at this stage. The upregulation of PYL4s and PYL8 suggests that in D, there are ABA-mediated responses, such as stomatal closure and inhibition of type 2 protein phosphatases (Hao et al., 2011). A previous study on tomatoes has shown that PP2C, when silenced, accelerates ABA-mediated fruit ripening (Zhang et al., 2018). This is relevant to our observations, since we also noted the downregulation of 15 and 5 PP2Cs in D, 60 and 90 DAF (Figure 3). Therefore, D ripening is also under the influence of ABA.

Noticeable was the exclusive expression of a gene associated with asymmetric cell division SHR in D3 peels. It is a TF that usually functions in quiescent center cells in the roots and causes asymmetric cell division, leading to radial pattern formation in roots and/or shoot axial organs (Dolan, 2001). Its expression in the D3 peels proposes that it might be associated with the observed phenotype. We say this because we also observed the increased expression of the two SCARECROW (SCR, *LOC18050540*, and *LOC18037167*) in D3 as compared with C4. Both the SCR and SHR genes act together to regulate asymmetric cell divisions (Schneijderberg, 2012). Another possible cause of the observed phenotypic variation between C and D fruits could be the exclusive expression of the CNR6 gene (*LOC18046579*) in C. The CNR6 gene has been previously implicated in the negative regulation of organs e.g., in maize (Guo et al., 2010). Thus, the exclusive expression of CNR6 in C3 could be related to a relatively smaller C fruit size as compared with D. The stomata differentiation gene (TF MUTE, *LOC18032141*) when not expressed (as in the case of D3) results in the abortion of meristemoids after their asymmetric division, resulting in no stomata differentiation (Pillitteri et al., 2007). Thus, it could also be one of the key differential changes in both peel types (Supplementary Table 3). Another possibility of differences in peel surface could be the exclusive expression of the GIL1 gene in D (Supplementary Table 3). We say this because GIL1 is required for light and phytochrome-mediated deregulation of negative gravitropism. This, in turn, causes a random hypocotyl growth orientation (Allen et al., 2006). A recent study has suggested that the reorientation in hypocotyl is possibly due to cell growth processes and auxin signaling (Adamowski et al., 2019). Since we observed the differential regulation of auxin-induced protein 15 (and 15-like) in C and D as well as cell division and cell number-related genes, therefore, the role of GIL1 is not negligible in the studied peel comparison. However, how these genes are interacting in lemon peels is yet to be explored.

Apart from the MAPK and plant hormone signaling pathways, the major differences were observed in ubiquinone and other terpenoid-quinone and terpenoid biosynthesis pathways, both in transcriptome and metabolome results (Supplementary Tables 4, 5). The downregulation of genes and reduced accumulation of most of the metabolites enriched in these pathways give an essential indication of the key biochemical differences in both peel types. Terpenoids are related to the aroma as well as fruit ripeness and quality (Fortes et al., 2011). Thus, their reduced biosynthesis in D could be one of the reasons for its aesthetic quality. These observations are very much important for traits other than peel roughness, such as aroma (Fei et al., 2021). It is important since the aroma is one of the key characteristics that shape consumer preferences. Of the other key differences, changes in cell wall composition seemed to be dominant since, in almost all of the treatment comparisons (except C5 vs. D4 in transcriptome), we observed the differential regulation of related genes and accumulation of related metabolites (Supplementary Tables 3–5). This observation is consistent with a previous study on the differential accumulation of cell wall-related metabolites and changes in cell wall thickness in mandarins (Lu et al., 2017). In this regard, the exclusive expression of XTH22 and XTH24 is quite relevant to the visible phenotype of D, i.e., thicker peel (Supplementary Tables 3, 5). These genes catalyze xyloglucan endohyrolysis (endotransglycosylation) and participate in cell wall construction in growing tissues (Xu et al., 1995; Nawaz et al., 2017). In contrast, the exclusive expression of coumarin 8-geranyltransferase 1b in C2 as compared with D1 could be causing increased coumarin content in C (Supplementary Table 5). This gene has already been characterized in *C. limon* (Munakata et al., 2014); it is a flavonoid-specific prenyltransferase in lemon and is specifically a prenyl donor for coumarins.

Finally, the increased expression of fatty acid elongation-related genes (elongation of fatty acids protein 3-like and A-like) and accumulation of metabolites classified as fatty acids in D are interesting for epicuticular wax roles (Supplementary Tables 3, 5). The fatty acid accumulation in the epicuticular covers the aerial parts and helps to protect the fruits against water loss and diffusion, and to withstand temperature fluctuations (Yeats and Rose, 2013). Thus, although the spring harvest has reduced aesthetics, it might have different abilities to withstand the aforementioned stresses. Furthermore, this knowledge is also useful for the overall improvement of lemon fruit quality. Finally, one of the major differences in gene expression and metabolite accumulation was the regulation of carotenoid biosynthesis and degradation. In the D peel, we observed the increased expression of probable carotenoid cleavage dioxygenase 4, while in the C peel, we observed the exclusive expression of geranylgeranyl pyrophosphate synthase 7 chloroplastic-like. These expression changes indicate that carotenoid biosynthesis in C is active, and that in the case of D, either carotenoid biosynthesis is reduced or degreening is active (Huang et al., 2009). Apart from carotenoids, both fruit peels also had differential anthocyanin biosynthesis, as evidenced by the decreased accumulation of leucoanthocyanidins (Supplementary Table 5). Thus, the color differences in both C and D fruits are largely due to the ethylene signaling and carotenoid and anthocyanin biosynthesis pathways.

### Future Directions

An important aspect of differential fruit development in D and C could be the temperature at the time of pollen and ovary formation, flowering, and early fruit formation in watermelon (Lin, 2007; Wei et al., 2015), tomato (Yang et al., 2009), and cucumber (Xiaoyan and Zhilong, 2008). Since the early growth period of the D fruits was in low temperatures (see material and methods section), therefore, the differences in fruit (particularly peel) development could be due to lower temperature. This assumption is based on earlier observation in longan where the fruits experiencing lower temperatures resulted in larger fruits due to its effects on cell division (Yang et al., 2010a,b). In this regard, earlier studies have shown the effect of cold on citrus leaves and different plant parts or plants as a whole have added much knowledge (Jian-huaa and Su-jinb, 2010; Jiang et al., 2016, 2021). However, the effect of cold on the differential peel formation in lemon is an open question for future studies. Specific experiments where lemon trees grown under controlled conditions could delineate the specific role(s) of temperature on the peel growth and variation. Not only, the cold temperature but the overall growth conditions (light, water, and nutrition, etc.) can also regulate the plant growth differently and might exert comprehensive influence on the regulation of genes and the accumulation of metabolites (Seymour et al., 2013). Thus, in further studies on the differences in lemon peels grown and harvested in different seasons, the other abiotic and biotic factors will help to further enhance our understanding of the mechanism of different peel traits.

## Conclusion

A combined transcriptome and metabolome analysis of the spring-harvested, rough-peeled and autumn-harvested, smooth-peeled *C. limon* explored the major pathways, genes, and metabolites that were exclusive and the differential between them. Our results propose the possible role of different genes related to asymmetric cell division, cell number regulation, randomization of hypocotyl growth, and stomata differentiation in peel roughening in lemon. The MAPK and plant hormone signaling pathways differentially regulated the peel development in early DAF. Particularly, our results suggest that ethylene signaling is playing a role in color differentiation in both peel types along with the carotenoid and anthocyanin biosynthesis pathways. Both peels differed in the accumulation of terpenoids, flavonoids, fatty acids, and lignans, and coumarins. The rough peels have higher fatty acid biosynthesis/elongation as compared the smooth peels. We also conclude that the thicker rough peels of spring-harvested lemon fruits are probably due to the increased expression of cell-wall related genes. Taken together, the rough peels have higher lignan and coumarin (cell-wall construction in growing tissues) and fatty acids, and reduced production of flavonoids and terpenoids. The effects of different abiotic and biotic factors on differential fruit peel development will further strengthen these conclusions.

## Materials and Methods

### Plant Material and Sampling

“Yunning No.1” lemon is mainly cultivated in tropic areas of Yunnan province, with sporadic cultivation in Sichuan, Guangxi, Guangdong, and Hainan provinces of China. Six-year-old grafted plants (disease- and insect-free) of *Citrus limon* (L.) Burm. f. “Yunning No.1” on *Poncirus trifoliata* L., growing at the Lujiangba Experimental base of the Institute of Tropical and Subtropical Economic Crops, Yunnan Academy of Agricultural Sciences (21°59′N, 98°53′E) were selected for fruit peel sampling. The experimental station lies in a subtropical dry-hot climate zone with an average annual temperature of 21.3°C (10-year average), average annual sunshine of 2,329.7 h, average radiation of 138,449 cal/(year.cm^2^), and an average rainfall of 755.3 mm. The quality of soil in the location is sandy loam with medium fertility. The experimental group consisted of lemon fruits that flowered in winter and ripened in spring. The samples were collected 60, 90, 120, and 150 days after flowering. The control group consisted of lemon fruits that flowered in spring and ripened in autumn. The samples were collected 30, 60, 90, 120, and 150 days after flowering (Figure 1). Since the temperatures in winter are low (on an average of 13.9°C), the peel and flesh are not very distinguishable, and it is not feasible to separate the peel in the case of the experimental group. However, the peel can be easily separated only after 60 days after flowering. Therefore, the sample at 30 days after flowering was not processed for the experimental group. The temperatures in both growing seasons are given in Supplementary Table 8. Three plants were selected for each time point, and triplicate samples were harvested for each time point in each season. The harvested fruits were surface-cleaned with distilled water and wiped to dry. Fruit peels were removed, wrapped in aluminum foil, and immersed and stored in liquid nitrogen.

### Transcriptome Sequencing

### RNA Extraction, cDNA Library Construction, and Transcriptome Sequencing

Ribonucleic acid extraction, cDNA library construction, and sequencing were performed at Novogene (Beijing NuoheZhiyuan Technology Co., Ltd). Briefly, total RNAs were extracted using the Spin Column Plant total RNA Purification Kit (Sangon Biotech, Shanghai, China); their purity was assessed on 1% agarose gels and quantified using an Agilent Bioanalyzer 2100 (Agilent Technologies, Sta. Clara, CA, United States) as reported earlier (Chen et al., 2019). Sequencing libraries were created using the NEB Next Ultra RNA Library Prep Kit following the instructions of the manufacturer. Twenty-four paired-end cDNA libraries with an insert size of 300 bp were constructed. The clustering of the index-coded samples was performed on a cBot Cluster Generation System using TruSeq PE Cluster Kit v3-cBot-HS (Illumina, San Diego, CA, United States) according to the instructions of the manufacturer. After cluster generation, the library preparations were sequenced on a Novaseq (Illumina, San Diego, CA, United States) platform, and 150 bp paired-end reads were generated.

### Data Analyses

Raw data (raw reads) of fastq format were first processed through in-house Perl scripts, and clean data (clean reads) were obtained. The checking of sequencing error rate distribution was performed according to Q_*phred*_ = −10log10 (e), where e = sequencing error rate and Q_*phred*_ = is the based quality value of Illumina. The Q20, Q30, and GC content of the clean data was calculated as reported earlier (Goldstein et al., 2016). All the downstream analyses were based on clean data with high quality.

An index of the reference genome was built using Hisat2 v2.0.5, paired-end clean reads were aligned to the reference genome (Xu et al., 2013; Kim et al., 2019; Li et al., 2020), and read numbers mapped to each gene were counted using the feature Counts v1.5.0-p3 (Liao et al., 2014). We then calculated the FPKM (fragments per kilobase of transcript sequence per millions base pairs sequenced) of each gene, and the differential expression analysis was performed using the DESeq2 R package (1.20.0). The *P*-values were adjusted using Benjamini and Hochberg’s approach (Benjamini and Hochberg, 1995) in order to control the FDR (false discovery rate). The threshold for differentially expressed genes (DEGs) was *P*-value < 0.05. The transcripts were then annotated in the Kyoto Encyclopedia of Genes and Genomes (KEGG) database (Kanehisa and Goto, 2000). We used the cluster Profiler R package to test the statistical enrichment of differential expression genes in KEGG pathways.

### qRT-PCR Analysis

Ten genes were randomly selected for verification of the transcriptome results through quantitative real-time PCR (qRT-PCR) reactions. We used gene-specific primers for qRT-PCR (Supplementary Table 7). The primers were designed in Primer3Plus (Untergasser et al., 2007). A housekeeping gene i.e., *Actin*, was used as an internal control. All the reactions were carried out in a Rotor-Gene 6000 (Qiagen, Shanghai, China) machine. The thermal cycles consisted of 50 (2 min) and 95°C (2 min) followed by 40 cycles of 95 (3 s) and 60°C (30 s). This was followed by the verification of single production amplification by melting curve analysis with temperatures ranging from 55 to 95°C by increasing 1°C every step. The total reaction volume was 10 μl (30 ng cDNA, 5 μl 1 × SYBR^®^ Select Master Mix (Applied Biosystem, Carlsbad, CA, United States), and.2 μl (20 μM) of each primer). We analyzed three biological replicates for each time point. The rest of the conditions for reactions and the analysis of the expression data were performed as reported earlier (Sendín et al., 2012; Chin et al., 2021).

### Metabolome Analysis

Tissues (100 mg) were separately ground with liquid nitrogen and mixed with 80% methanol and.1% formic acid. The mixture was incubated on ice for 5 min and then centrifuged at 15,000 *g* for 20 min at 4°C. The supernatant was diluted to a final concentration containing 53% methanol by adding LC-MS grade water. The solution was again centrifuged at 15,000 *g* for 20 min at 4°C, and the resulting supernatant was used for liquid chromatography with tandem mass spectrometry (LC-MS/MS) (Want et al., 2006).

The ultra-high performance (UHP) LC-MS/MS analysis was conducted at Novogene Co., Ltd. (Beijing Novogene Technology Co., Ltd)^1^ following their standard procedures. The raw data files obtained by UHPLC-MS/MS analyses were processed using the Compound Discoverer 3.1 (CD3.1; Thermo Fisher Scientific, Waltham, MA, United States) to perform peak alignment, peak picking, and quantitation for each metabolite. Briefly, the data were screened by retention time and mass-to-charge ratio. To make the metabolite identification accurate, peak alignment was performed according to retention time deviation and mass deviation (part per million, ppm). Following this, signal-to-noise ratio, adduct ion, and peak area were quantified in ppm. We then identified the metabolites by comparing the quantified data with mzCloud, mzVault, and the MassList primary database search library. Metabolites with a coefficient of variation less than 30% (Dai et al., 2017) in the QC sample were retained as the final identification result for subsequent analyses (Fernie et al., 2011).

Statistical analyses were performed using the statistical software R (R version R-3.4.3), Python (Python 2.7.6 version), and CentOS (CentOS release 6.6); when data were not normally distributed, normal transformations were attempted using area normalization method.

Based on the total putative identified metabolites as background in each pathway, we performed a hypergeometric test to find the significantly enriched KEGG pathways for the differentially accumulated metabolites with the R software. The *p*-values were adjusted by FDR with a threshold of less than or equal to.05.

The identified metabolites were annotated using the KEGG^2^ (Kanehisa and Goto, 2000), HMDB^3^ (Wishart et al., 2018), and LIPIDMaps databases^4^. The principal component analysis (PCA) and partial least squares discriminant analysis (PLS-DA) were performed in metaX (Boulesteix and Strimmer, 2007; Wen et al., 2017). We performed a univariate analysis (*t*-test) to calculate the statistical significance (*P*-value). The metabolites with VIP > 1 and *P* < 0.05 and fold change ≥ 2 or FC ≤ 0.5 were considered to be differentially accumulated metabolites (DAMs). Volcano plots were used to filter metabolites of interest, which were based on log2 FC and −log10 (*p*-value) of the metabolites.

For clustering heatmaps, the data were normalized using z-scores of the intensity areas of differential metabolites and were plotted with the Pheatmap package in the R language. The Pearson’s correlation between differential metabolites was analyzed by cor in the R language. *P*-value < 0.05 was considered as statistically significant, and correlation plots were plotted with the corrplot package in the R language. The functions of these metabolites and metabolic pathways were studied using the KEGG database. The metabolic pathway enrichment of differential metabolites was performed; when ratio was satisfied by x/n > y/N, a metabolic pathway was considered as enrichment; when the *P*-value of a metabolic pathway < 0.05, the metabolic pathway was considered as statistically significant enrichment.

## Data Availability Statement

The original contributions presented in the study are publicly available. This data can be found here: National Center for Biotechnology Information (NCBI) BioProject database under accession number PRJNA716747.

## Author Contributions

H-ML, C-RL, J-XL, and F-GH: conceptualization. H-ML, C-RL, S-HW, X-MF, X-YZ, J-MM, H-XY, and Y-XD: methodology. J-QY: software. H-ML and C-RL: validation, formal analysis, and data curation and writing—original draft preparation. H-ML, C-RL, S-HW, X-MF, X-YZ, J-MM, H-XY, and Y-XD: investigation. H-ML, J-XL, and F-GH: resources. J-XL and F-GH: writing—review, editing, and project administration. All authors contributed to the article and approved the submitted version.

## Conflict of Interest

The authors declare that the research was conducted in the absence of any commercial or financial relationships that could be construed as a potential conflict of interest.

## Publisher’s Note

All claims expressed in this article are solely those of the authors and do not necessarily represent those of their affiliated organizations, or those of the publisher, the editors and the reviewers. Any product that may be evaluated in this article, or claim that may be made by its manufacturer, is not guaranteed or endorsed by the publisher.

## Footnotes

1
https://en.novogene.com/

2
https://www.genome.jp/kegg/pathway.html

3
https://hmdb.ca/metabolites

4
http://www.lipidmaps.org/
