# Supplementary material for: Transcriptome and Metabolome Comparison of Smooth and Rough Citrus limon L. Peels Grown on Same Trees and Harvested in Different Seasons
Source: Front Plant Sci. 2021 Oct 8;12:749803. doi: 10.3389/fpls.2021.749803 (PMC8531254; doi:10.3389/fpls.2021.749803)
Supplement: Supplementary file 2 [file Data_Sheet_2.docx]

**Supplementary Figure 1.** A) Distribution of sample gene expression and B) Pearson correlation coefficients in *Citrus limon* fruit peels in different treatment comparisons. C1, C2, C3, C4, and C5 represent samples collected 30, 60, 90, 120, and 150 days after flowering. D1, D2, D3, and D4 represent samples collected 60, 90, 120, and 150 days after flowering. The numbers 1, 2, and 3 with each treatment represent the three replicates.


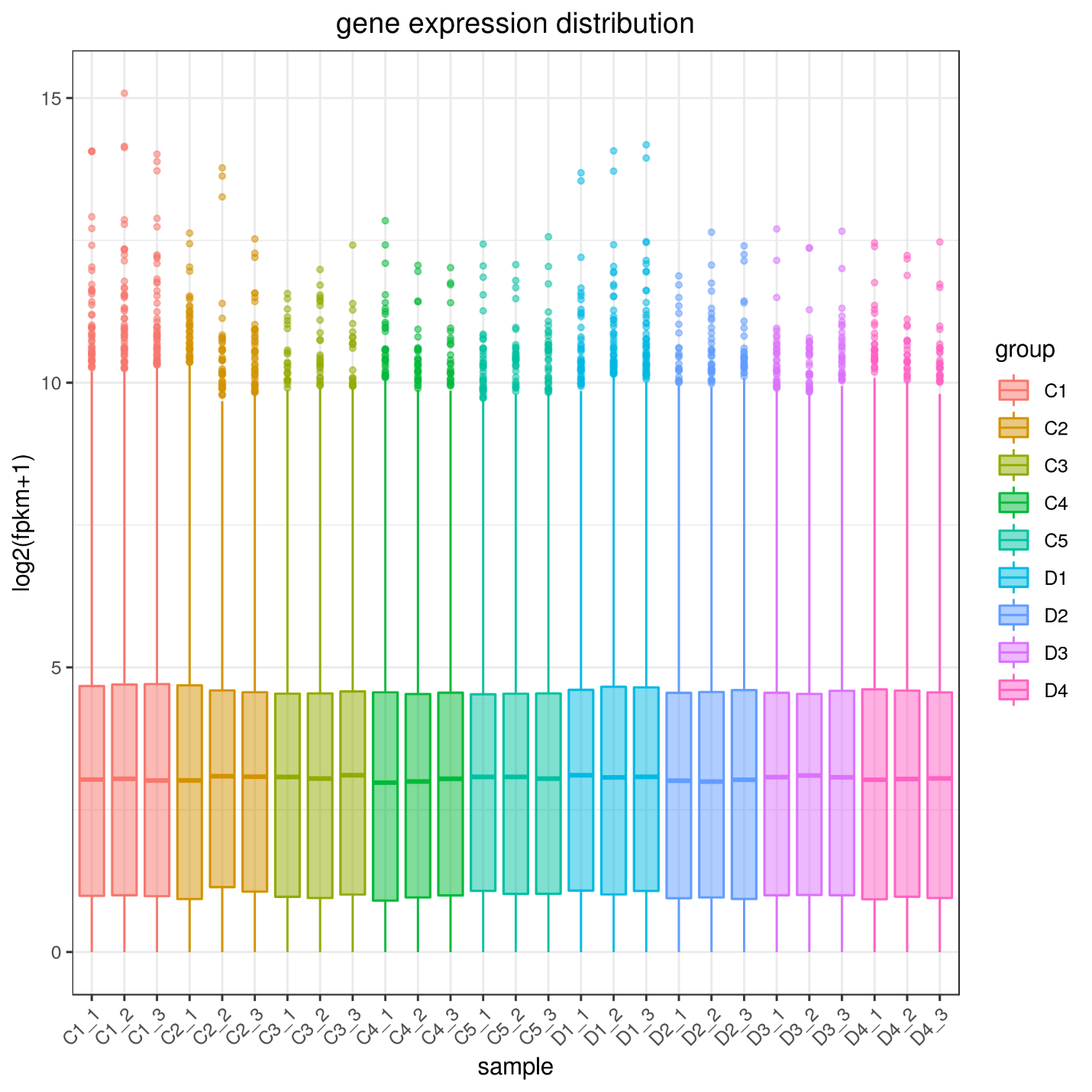


A


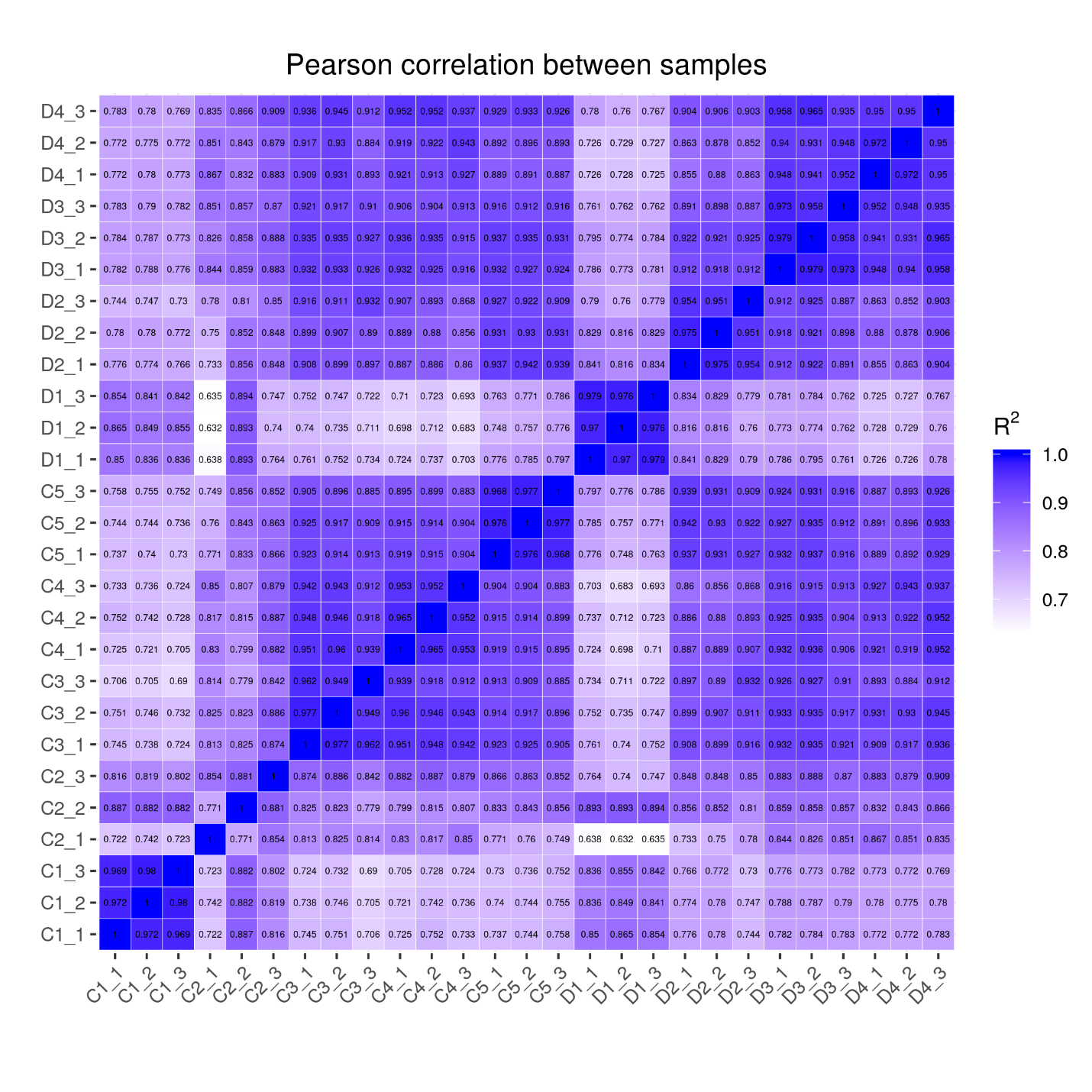


B

**Supplementary Figure 2.** Enrichment of differentially expressed genes in a) C2 vs D1, b) C3 vs D2, c) C4 vs D3, and d) C5 vs D4 in KEGG pathways. C2, C3, C4, and C5 represent samples collected 60, 90, 120, and 150 days after flowering. D1, D2, D3, and D4 represent samples collected 60, 90, 120, and 150 days after flowering.


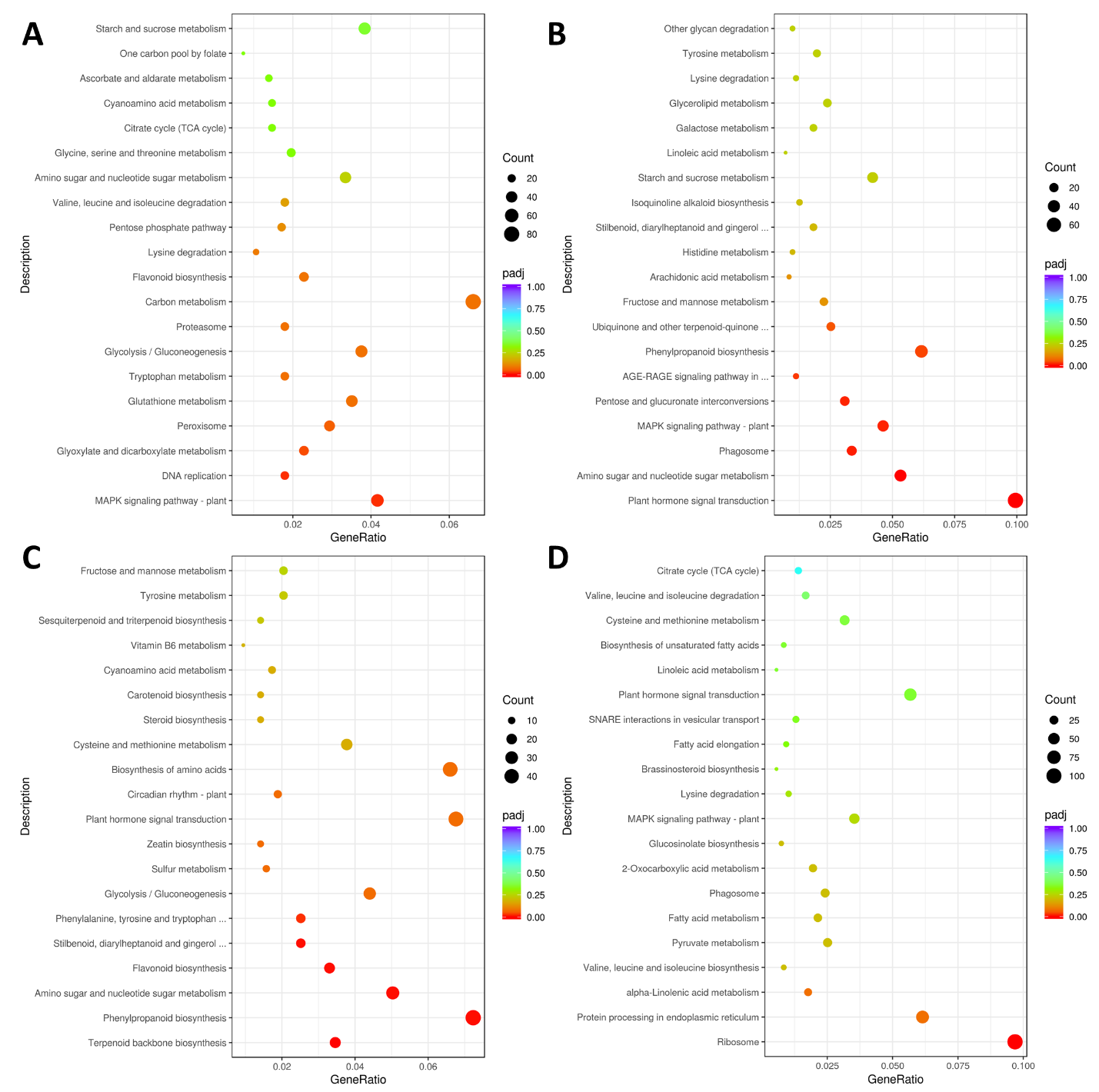


**Supplementary Figure 3.** Enrichment of differentially accumulated metabolites in C and D type *C. limon* peels. C2, C3, C4, and C5 represent samples collected 60, 90, 120, and 150 days after flowering. D1, D2, D3, and D4 represent samples collected 60, 90, 120, and 150 days after flowering.
